# The Presence of TGFβ3 in Human Ovarian Intrafollicular Fluid and Its Involvement in Thromboxane Generation in Follicular Granulosa Cells through a Canonical TGFβRI, Smad2/3 Signaling Pathway and COX-2 Induction

**DOI:** 10.3390/ijms25105558

**Published:** 2024-05-20

**Authors:** Tsung-Hsuan Lai, Hsuan-Ting Chen, Pi-Hui Wu, Wen-Bin Wu

**Affiliations:** 1 Department of Obstetrics and Gynecology, Cathay General Hospital, No. 280, Renai Rd. Daan Dist., Taipei 10693, Taiwan; joseph@cgh.org.tw; 2 School of Medicine, Fu Jen Catholic University, No. 510, Zhongzheng Rd., Xinzhuang Dist., New Taipei City 242062, Taiwan; 713008.apple@yahoo.com.tw; 3 Ph.D. Program in Pharmaceutic Biotechnology, Fu Jen Catholic University, No. 510, Zhongzheng Rd., Xinzhuang Dist., New Taipei City 242062, Taiwan; jenny123142003@gmail.com; 4 Graduate Institute of Biomedical and Pharmaceutical Science, Fu Jen Catholic University, No. 510, Zhongzheng Rd., Xinzhuang Dist., New Taipei City 242062, Taiwan

**Keywords:** transforming growth factor, TXA_2_, cyclooxygenase, COX, IVF, oocyte maturation

## Abstract

Ovarian follicular fluid (FF) has a direct impact on oocyte quality, playing key roles in fertilization, implantation, and early embryo development. In our recent study, we found FF thromboxane (TX) to be a novel factor inversely correlated with oocyte maturation and identified thrombin, transforming growth factor β (TGFβ), TNF-α, and follicular granulosa cells (GCs) as possible contributors to FF TX production. Therefore, this study sought to investigate the role of TGFβ3 in regulating TX generation in human ovarian follicular GCs. TGFβ3 was differentially and significantly present in the FF of large and small follicles obtained from IVF patients with average concentrations of 68.58 ± 12.38 and 112.55 ± 14.82 pg/mL, respectively, and its levels were correlated with oocyte maturity. In an in vitro study, TGFβ3 induced TX generation/secretion and the converting enzyme-COX-2 protein/mRNA expression both in human HO23 and primary cultured ovarian follicular GCs. While TGFβRI and Smad2/3 signaling was mainly required for COX-2 induction, ERK1/2 appeared to regulate TX secretion. The participation of Smad2/3 and COX-2 in TGFβ3-induced TX generation/secretion could be further supported by the observations that Smad2/3 phosphorylation and nuclear translocation and siRNA knockdown of COX-2 expression compromised TX secretion in GCs challenged with TGFβ3. Taken together, the results presented here first demonstrated that FF TGFβ3 levels differ significantly in IVF patients’ large preovulatory and small mid-antral follicles and are positively associated with oocyte maturation. TGFβ3 can provoke TX generation by induction of COX-2 mRNA/protein via a TGFβR-related canonical Smad2/3 signaling pathway, and TX secretion possibly by ERK1/2. These imply that TGFβ3 is one of the inducers for yielding FF TX in vivo, which may play a role in folliculogenesis and oocyte maturation.

## 1. Introduction

In vitro fertilization (IVF) is conducted for the treatment of infertility by using several different stimulation protocols [1], and the success rate of IVF largely depends on oocyte quality. Follicular fluid (FF) is a liquid that fills the follicular antrum and surrounds the ovum in an ovarian follicle, which contains steroid hormones, substances, and many metabolites and can be critical for oocyte growth and development [2]. The formation of the antrum with FF in a typical follicle can be an antral follicle or preovulatory follicle, which also segregates the granulosa cells (GCs) with respect to the primary oocyte into two specific regions: the cumulus GC region and the mural GC region lining the wall of the follicle [3]. More importantly, FF has a direct impact on oocyte quality, playing key roles in fertilization, implantation, and early embryo development [4,5,6].

Prostaglandins (or prostanoids; PGs) are a group of C20 lipid mediators (eicosanoids) synthesized from arachidonic acid (AA) by a key enzyme, namely cyclooxygenase (COX). The AA-COX pathway produces five bioactive metabolites consisting of four kinds of PGs and thromboxane (TX), which are PGE_2_, PGD_2_, PGF_2_, PGI_2_, and TXA_2_ [7]. These PGs, especially PGE_2_ and PGF_2α_, have clinically relevant roles in reproductive biology, including promoting ovulation downstream of the luteinizing hormone surge and corpus luteum development [8]. It has been reported that excess consumption of NSAIDs, which inhibit COX, is associated with reversible female infertility, likely due to failed ovulation [9]. In addition, mice deficiency in COX-2 showed multiple reproductive failures during early pregnancy such as failures in ovulation, fertilization implantation, and decidualization [10], and abnormal COX-2 levels are associated with ovulation failure, infertility, and implantation disorders [11,12].

The role of PGE_2_ and PGF_2α_ has been extensively reported in early reproductive processes such as ovulation, luteolysis, and fertilization in domestic animals like sheep and cows [7]. It has been reported that a large amount of PGE_2_ is released into the FF [13], and PGE_2_-EP_2_ signaling stimulates cumulus ECM disassembly [7]. Accordingly, the COX-2 null mice and mice lacking the PGE EP_2_ receptor (which is mainly expressed in the cumulus) show severely impaired and reduced ovulation [14,15], suggesting that PGE_2_-EP_2_ signaling plays roles in ovulatory processes [7].

Thromboxane A_2_ (TXA_2_) is a prostanoid originally involved in platelet aggregation that mainly participates in hemostasis. Our previous study showed that the plasminogen activator inhibitor-1 that participates in hemostasis is differentially expressed in the FF of large and small follicles of IVF patients [16]. In addition, our recent investigation showed that a significant amount of TXB_2_, a stable metabolite of TXA_2_, is present in the FF of IVF patients’ follicles, and its expression level is inversely related to the sizes of those patients’ follicles and is negatively correlated with oocyte maturation. Further analyses revealed that thrombin, a clotting factor IIa that is involved in blood coagulation, is also present in IVF patients’ follicles and can act as an inducer for TX production and secretion in follicular GCs through PAR-2 and downstream MAPK-related cellular signaling pathways. More interestingly, in that study, we demonstrated that thrombin and TX appear to participate in follicle development in an ex vivo ovary culture model, which promotes ovarian follicle development and growth rate [17]. Therefore, FF TX is highly suspected as a factor participating in folliculogenesis and oocyte maturation in the female reproduction system. 

Transforming growth factor β (TGFβ) is a prototype of the TGFβ family [18]. Currently, three subtypes of TGF-β (TGFβ1-3) are found to be expressed in mammals. As a multifunctional polypeptide cytokine, TGF-β plays a critical role in early embryonic development and adult homeostasis [19]. All cells in the developing embryo and the adult can perceive TGFβ family signals and respond with effects on cell proliferation, differentiation, communication, adhesion, movement, metabolism, and death that are as varied as the target cell types [20]. The low-affinity heteromeric receptor complex (tβR I with tβR II) conducted by activated TGFβ stimulates different downstream signaling pathways, including canonical Smad pathways and non-canonical no-Smad pathways to regulate context-dependent transcription [21]. In the female reproductive system, it has been reported that TGFβ1 and -2 stimulate FSH receptor expression, TGFβ1 synergizes with VEGF to promote angiogenesis, TGFβ1 inhibits estradiol production, TGFβ3 promotes follicle development, and TGFα upregulates IL-8 expression at different stages of folliculogenesis and luteinization [22]. Moreover, a positive correlation of FF TGFβ and progesterone levels with the expression of COX-2 and hyaluronic acid synthase 2 in cumulus cells suggests that FF TGFβ can predicts a better follicle quality [23]. 

As mentioned above, FF TX is associated with oocyte maturation and suspected of being a mediator participating in follicle development. In addition, TGFβ3 acts as a possible inducer for TX generation by ovarian follicular GCs in a preliminary screening in our parallel study [17]. Therefore, this study further investigates how TGFβ3 affects and regulates TX production in ovarian follicular GCs. Our results indicated that TGFβ3 was able to cause TXB_2_ generation and secretion in GCs through COX-2 induction, which was mediated by activation of the canonical Smad2/3-dependent signaling pathway.

## 2. Results

### 2.1. The TGFβ3 Is Present in the Ovarian FF of IVF Patients and Its Levels Are Positively Correlated with Oocyte Maturity

**Figure 1:**
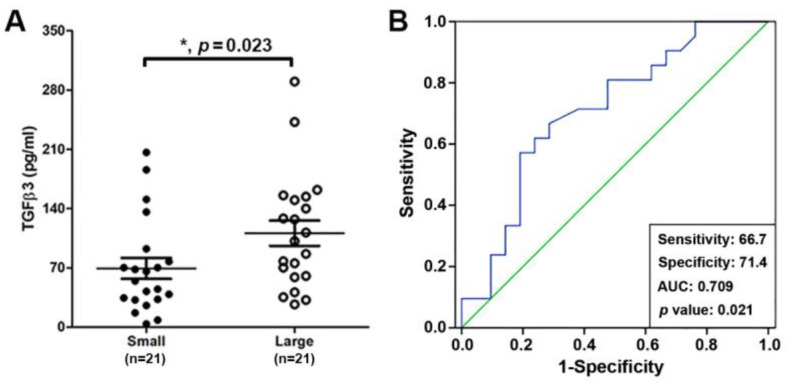
Ovarian FF TGFβ3 levels are positively correlated with oocyte maturity. (**A**) The scatter plot and paired Student’s *t*-test of FF TGFβ3 levels between a large (preovulatory) leading follicle (diameter > 18 mm) and small (mid-antral) follicles. (**B**) The correlation of FF TGFβ3 levels and oocyte maturity was performed by receiver operating characteristic (ROC) curve analysis (* *p* < 0.05).

Our recent study showed that thrombin, TGFβ3, and TNF-α can cause TX production in human primary cultured ovarian follicular GCs in an in vitro preliminary screening assay [17]. In this study, we first evaluated whether TGFβ3 is present in human ovarian FF. The TGFβ3 expression levels in individual FF samples of a large (preovulatory) leading follicle (diameter > 18 mm) and a small (mid-antral) follicle (diameter < 14 mm) from each IVF patient during oocyte retrieval were measured by ELISA. It was found that the average concentrations of TGFβ3 in small and large follicles were 68.58 ± 12.38 and 112.55 ± 14.82 pg/mL, respectively. The scatter plot and paired Student’s *t*-test of these data revealed a significant difference in FF TGFβ3 levels between the two groups (*p* = 0.023 < 0.05, *n* = 21) (Figure 1A). To further identify the correlation between TGFβ3 levels and oocyte maturity, logistic analysis and receiver operating characteristic (ROC) curves analysis were performed. The results indicated a significant association between the TGFβ3 levels and oocyte maturity in logistic analysis (*p* = 0.039 < 0.05) (Table 1) and FF TGFβ3 levels were correlated with oocyte maturity (AUC = 0.702, sensitivity =66.7%, specificity = 71.4%, *p* = 0.021 < 0.05) (Figure 1B). Finally, the Spearman rank correlation analysis revealed a significantly positive correlation between FF TGFβ3 levels and oocyte maturity with a correlation coefficient of 0.353 (*p =* 0.022 < 0.05).

### 2.2. TGFβ3 Promotes COX-2 but Not COX-1 Expression in Human HO23 GC Cell Line and Primary Cultured Ovarian Follicular GCs

**Figure 2:**
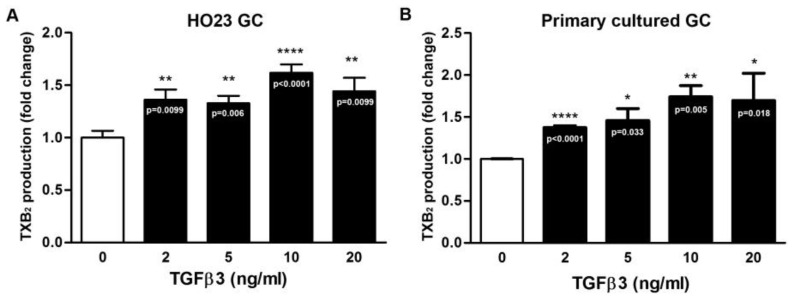
TGFβ3 increases TXB_2_ production in (**A**) human HO23 GCs and (**B**) primary cultured ovarian follicular GCs. GCs were treated with the indicated concentrations of TGFβ3 for 24 h. The culture media were collected and immediately analyzed for TXB_2_ production by ELISA (*n* = 3). * *p* < 0.05, ** *p* < 0.01, and **** *p* < 0.0001 versus vehicle only (control; TGFβ3: 0 ng/mL) and the exact *p*-values for all significant differences are shown in each bar.

**Figure 3:**
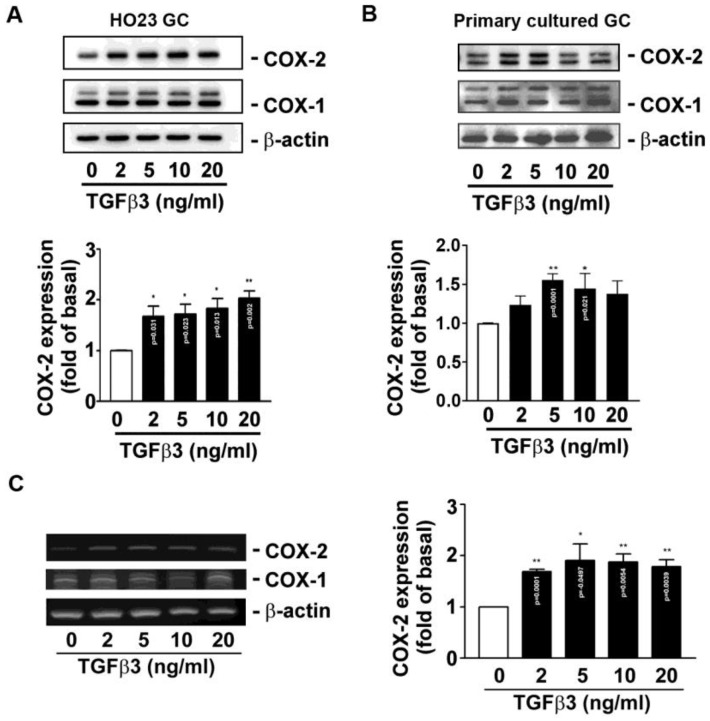
TGFβ3 enhances COX-2 protein and mRNA expression in human HO23 GCs and primary cultured ovarian follicular GCs. (**A**) Human HO23 GCs or (**B**) human primary cultured ovarian follicular GCs were treated with the indicated concentrations of TGFβ3 for 24 h, and COX-1 and COX-2 protein expression were analyzed by Western blotting. (**C**) Human HO23 GCs were treated with the indicated concentrations of TGFβ3 for 4 h. At the end of incubation, cells were collected, total RNA was extracted, and the COXs and β-actin mRNA were analyzed by RT-PCR. Data are expressed as mean ± SEM (*n* = 3). * *p* < 0.05 and ** *p* < 0.01 versus vehicle only (control; TGFβ3: 0 ng/mL) and the exact *p*-values for all significant differences are shown.

To further explore whether TGFβ3 affects TXB_2_ production and secretion, ELISA was performed. The human HO23 follicular GC cell line and primary cultured ovarian follicular GCs were used as the main materials. In Figure 2, TGFβ3 induced TXB_2_ production both in the human HO23 follicular GC cell line and primary cultured ovarian follicular GCs. In line with this observation, Western blotting of COX expression indicated that TGFβ3 provoked COX-2 protein expression in the human HO23 follicular GC cell line in a concentration-dependent manner. Similarly, TGFβ3 also induced COX-2 protein expression in primary cultured ovarian follicular GCs. In contrast, the COX-1 protein expression was not affected in both cells (Figure 3A,B). 

Because a similar induction COX-2 protein profile was exhibited in these two cells, the human HO23 cells were used as the main cells during this study due to limited primary cultured GCs. The RT-PCR analysis was performed to assay the *cox* mRNA expression in HO23 cells. It was found that COX-2, but not COX-1 mRNA, was upregulated in HO23 GCs’ response to TGFβ3’s challenge (Figure 3C). 

### 2.3. Participation of TGFβRI, Smad3, COX-2, and MAPK in TGFβ3-Induced TXB_2_ Production

**Figure 4:**
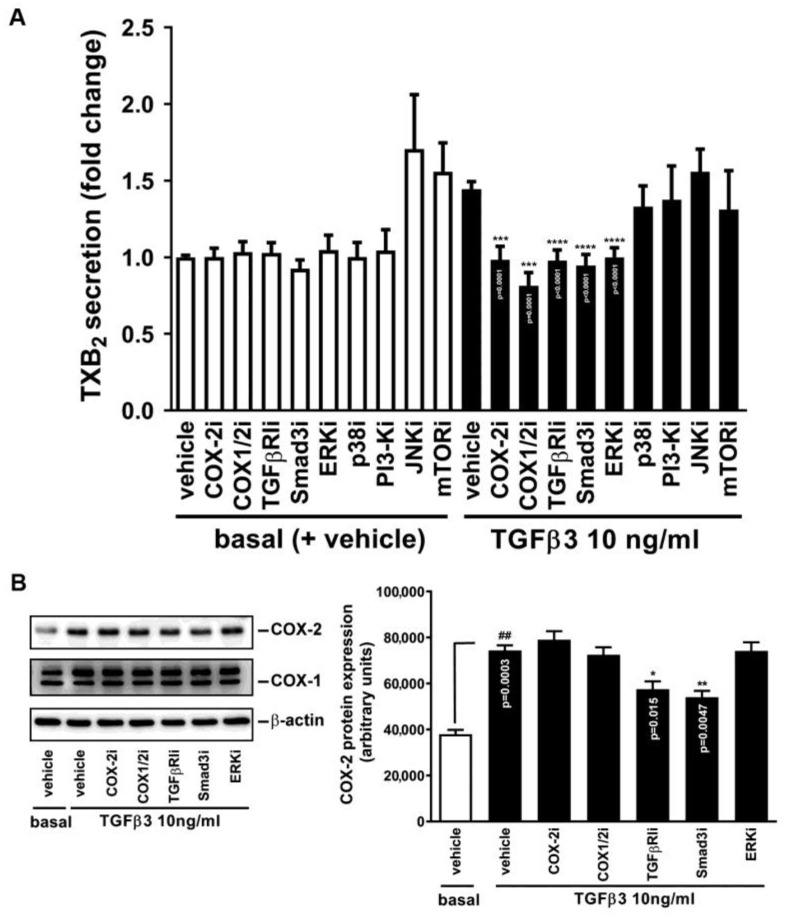
Effect of pharmacological interventions on TGFβ3-induced TXB_2_ generation and COX-2 expression. Human HO23 GCs were treated with vehicle or TGFβ3 (10 ng/mL) with or without the indicated inhibitor for 24 h. (**A**) The culture media were collected and immediately analyzed for TXB_2_ production by ELISA. (**B**) The COX-1 and -2 expressions were analyzed by Western blotting and quantitated by densitometry (*n* = 3–6). I: inhibitor. ^##^ *p* < 0.01 versus basal level, * *p* < 0.05, ** *p* < 0.01, *** *p* < 0.001, **** *p* < 0.0001 versus TGFβ3 control (—, +vehicle) and the exact *p*-values for all significant differences are shown.

For a detailed understanding of the mechanism of action of TGFβ3 in causing TXB_2_ production, pharmacological interventions toward cellular signaling were used to dissect the role of the involved signaling molecule(s). These inhibitors were LY364947 for TGFβRI [denoted as TGFβRIi], SIS3 for Smad3 (Smad3i), NS398 and indomethacin for COX-2 and/or COX-1(COX-2i and COX1/2i for each), PD98059 for ERK1/2 (ERKi), SB2020190 for p38 MAPK (p38i), SP600125 for JNK1/2 (JNKi), and rapamycin for mTOR (mTORi). In Figure 4A, the TXB_2_ secretion was significantly reduced in the presence of TGFβRIi, Smad3i, COX-2i, COX1/2i, and ERKi, highly suggesting that TGFβRI, Smad3, COX-2, and ERK1/2 mediated TGFβ3-provoked TXB_2_ production in GCs (Figure 4). 

Meanwhile, to further trace whether the above-identified cellular signaling components are necessarily required for TGFβ3-induced COX-2 expression, Western blotting was performed. In Figure 4B, only TGFβRIi and Smad3i attenuated TGFβ3-induced COX-2 expression. There was no doubt that NS398 (COX-2i) and indomethacin (COX1/2i), the compounds known to interfere with COX by affecting its activity but not expression, exhibited no inhibitions toward TGFβ3-induced COX-2 expression. Interestingly, ERKi was also shown to be ineffective against COX-2 induction, suggesting that ERK1/2 activation is involved after COX-2 induction and may participate in the thromboxane secretory process.

### 2.4. Direct Application of TGFβ3 to GCs Leads to Smad2/3 Phosphorylation and Translocation from Cytosol to Nucleus

**Figure 5:**
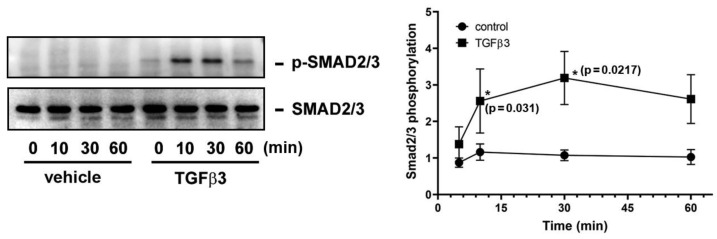
TGFβ3 induces Smad2/3 phosphorylation in GCs. Human HO23 GCs were treated with vehicle or TGFβ3 (10 ng/mL) for the indicated time intervals. The Smad2/3 phosphorylation and its total protein expression were analyzed by Western blotting and densitometry (*n* = 4). * *p* < 0.05 compared to vehicle treatment at the corresponding time point (15 and 30 min) and the exact *p*-values for the significant differences are shown.

In Figure 4, SIS3 as a Smad3 inhibitor blocks TGFβ3-provoked TXB_2_ production and COX-2 expression in GCs; therefore, WB analysis was performed to examine whether TGFβ3 treatment can directly induce Smad2/3 activation. Indeed, TGFβ3 significantly enhanced Smad2/3 phosphorylation in the cultured GCs. The Smad2/3 activation was significantly increased at 15 and 30 min and declined thereafter. In contrast, its total protein remained unchanged upon TGFβ3 treatment (Figure 5).

**Figure 6:**
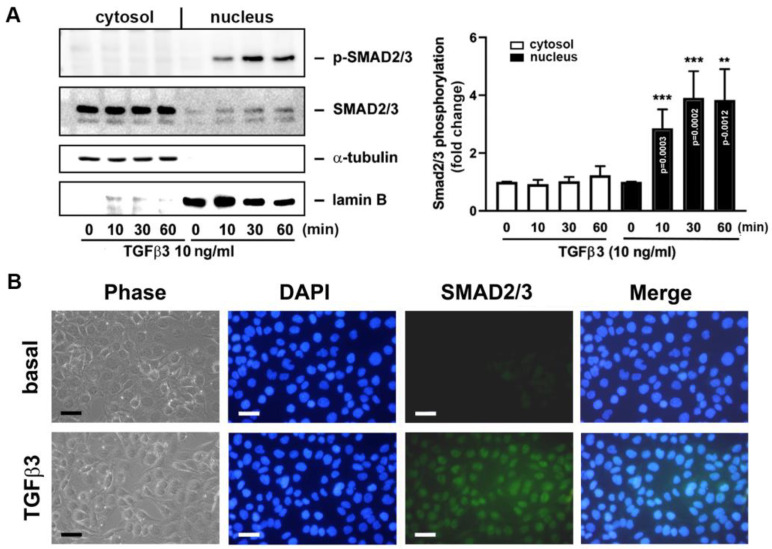
TGFβ3 induces Smad2/3 translocation from cytosol to the nucleus. Human HO23 GCs were treated with vehicle or TGFβ3 (10 ng/mL) for the indicated time intervals. (**A**) The cell lysates were fractionated as a cytosolic and nuclear fraction, and p-Smad2/3 and total Smad2/3 expressions in cytosolic and nuclear compartments were determined by Western blotting and densitometry (*n* = 5). ** *p* < 0.01 and *** *p* < 0.001 versus its corresponding controls (vehicle treatment at 10, 30, and 60 min) and the exact *p*-values for the significant differences are shown. (**B**) The Smad2/3 translocation was assayed by immunofluorescence microscopy. Merge: an overlay of images of Smad2/3 staining with DAPI staining. Scale bar = 50 μm.

It has been reported that Smad2/3 can translocate into the cell nucleus once they are activated and act as a transcription factor (TF) to drive specific gene transcription [21]. Next, we determined if the activated Smad2/3 can enter the cell nucleus upon TGFβ3 stimulation. In Figure 6A, the cytosolic and nuclear fractions were prepared and the Smad2/3 expression and phosphorylation in both compartments were assayed by Western blotting and densitometry. It was noted that Smad2/3 expression was fairly increased in the cell nucleus when cells were treated with TGFβ3. However, the phosphorylated form of Smad2/3 was apparently increased in the cell nucleus after 10 min, reaching the peak and plateauing at 30 and 60 min. The Smad2/3 translocation to the cell nucleus was further confirmed by immunofluorescence microscopy with anti-Smad2/3 Ab and DAPI, which could detect Smad2/3 and the cell nucleus, respectively. It was shown that the Smad2/3 staining was markedly enhanced in the nucleus when cells were challenged with TGFβ3 (Figure 6B).

### 2.5. Requirement of COX-2 Induction for TGFβ3-Mediated TXB_2_ Production

**Figure 7:**
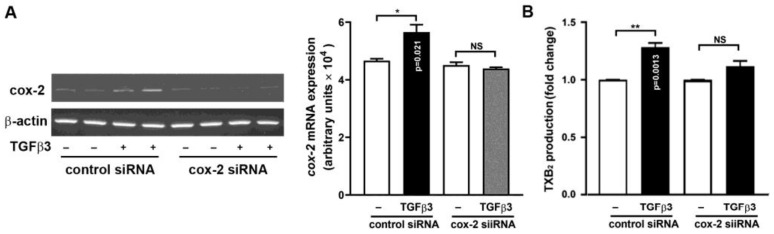
SiRNA knockdown of COX-2 expression compromises TGFβ3-induced TXB_2_ production. The HO23 GCs were transfected with control or COX-2 siRNA (150 or 300 nM) for 24 h and followed by the addition of vehicle or TGFβ3 (10 ng/mL) for 4 h. The cells were collected, (**A**) total RNA was extracted, and COX-2 mRNA expression was determined by RT-PCR, whereas (**B**) the culture media were collected and TGFβ3-induced TXB_2_ production was measured by ELISA. * *p* < 0.05 and ** *p* < 0.01 versus basal (control or COX-2 siRNA transfection with vehicle treatment only) and the exact *p*-values for the significant differences are shown. NS: not significant.

To ensure COX-2 is required for TGFβ3-induced TXB_2_ production, the small interference RNA (siRNA) was introduced to specifically knockdown (KD) COX-2, and ELISA was performed to assay the TGFβ3-induced TXB_2_ production. In Figure 7A, the COX-2 KD by siRNA led to a substantial decrease in COX-2 mRNA expression, demonstrating the COX-2 siRNA’s specificity. Concomitantly, the TGFβ3-induced TXB_2_ production was inhibited (Figure 7B), indicating a requirement of COX-2 for TGFβ3-induced TXB_2_ production.

## 3. Discussion

COX-2 deficiency in mice shows multiple reproductive failures during early pregnancy, such as failures in ovulation, fertilization implantation, and decidualization [10]. Moreover, uncontrolled inflammation has a significant impact on female reproduction such as endometriosis, polycystic ovary syndrome (PCOS) genesis, implantation, pregnancy, and labor [11]. In this study, we showed that TGFβ3 could enhance TX generation and secretion in ovarian follicular GCs, suggesting that GCs can produce TX in female reproductive follicle cells. One may be concerned that some of the results were obtained from the follicular HO23 GC cell line. To this end, we show that TGFβ3 caused a similar induction profile on TXB_2_ production and COX-2 protein expression in both HO23 and human primary cultured GCs, demonstrating that the GC cell line can be representative of this study. 

The main role of TXA_2_ was previously restricted to platelet aggregation, vasoconstriction, and smooth muscle cell mitogen [24,25]. However, it was shown recently that TXA_2_ can also be released by a variety of cells such as macrophages, neutrophils, and endothelial cells [26]. Although some studies reported that the stable TXB_2_ metabolite of TXA_2_ is present in FF [27,28,29], its function in the female reproduction system remains unclear. Our recent results demonstrated that FF TX levels were strongly correlated with oocyte maturation in human IVF patients and the TP receptor activated by a TXA_2_ analog (-U46619) regulates follicle development. Moreover, ovarian follicular GCs can be a source for TX production in ovarian FF [17]. In this study, TGFβ3 at 2-10 ng/mL was able to induce TXB_2_ production and secretion. This result, together with the finding of TGFβ3 presence in human FF, reveals that FF TGFβ3 could be used as one of the inducers responsible for FF thromboxane production. To our knowledge, this is the first study to show that TGFβ3 could enhance TXB_2_ production in ovarian follicular GCs through COX-2 induction.

Regarding the presence of TGFβ3 in the female reproduction system, it has been reported that in sheep, mRNA encoding both TGFβ1 and -2 is synthesized by ovarian theca, stroma, and cells of the vascular system, whereas TGFβ3 mRNA is synthesized by vascular cells. The authors suggest that TGFβ be potentially taken as an important autocrine regulator of thecal cell function and possibly a paracrine regulator of ovarian cell function at various development stages [30]. The steroidogenic factor-1 is required for TGFβ3-mediated 17β-estradiol synthesis in mouse ovarian GCs [31]. Moreover, expression of TGFβ3 is found in the porcine ovary during the estrus cycle [32]. Finally, an increased TGFβ1, -β2, and -β3 protein pattern is observed in oocytes of large compared to small follicles. The highest GDF9 and TGFβ1 mRNA levels are found in oocytes after in vitro maturation (IVM) compared to those before IVM. Based on that study, the authors supposed that the distribution pattern of TGFβ superfamily genes is associated with the stage of maturation of porcine oocytes and the follicle size [33]. In human beings, when antral follicles develop, TGFβ3 is the most abundant TGFβ isoform, and TGFβ1 protein levels decline in large follicles [34]. TGFβ1 and TGFβ3, but not TGFβ2, are upregulated in the ovaries of ovarian hyperstimulation syndrome [35]. In this study, the paired Student’s *t*-test showed a statistical significance of FF TGFβ3 levels between small and large follicles of IVF patients, with an average TGFβ3 level of 68.58 ± 12.38 and 112.55 ± 14.82 pg/mL in small and large follicles, respectively. In contrast to TGFβ3, TGFβ1 can be detected in the ovarian FF and ranges from about 400 to 2000 pg/mL among three IVF patient groups with different patient’s serum anti-Müllerian hormone levels [36]. The concentrations in FF were less than we used in the in vitro cell experiments. Based on the efficacy of TGFβ3 in causing COX-2 expression and TXB_2_ production, TGFβ3 appears to act as a moderate inducer for TX production in FF. 

There are some FF components reported to correlate with oocyte maturation [37], including angiotensin-(1–7), GDF-9, and lysophosphatidic acid [38,39,40]. More recently, metabolomic analysis indicates that the vitamin D3 pathway is strongly associated with ovarian stimulation outcomes in FF [41], and mitochondrial transcription factor A expression in follicular GCs is implicated in oocyte maturation [42]. We revealed here that TGFβ3 was also positively correlated with oocyte maturation, as assayed by the ROC curve and Spearman rank coefficient analysis. In this regard, this positive correlation seems to contradict our parallel study, which shows that FF TX levels are inversely associated with oocyte maturation [17]. We thus suggest that FF TGFβ3 is a moderately acting factor for TX production in GCs and may possess multiple facets in affecting oocyte maturation.

In this study, the cellular signaling responsible for TGFβ3-induced TX production was acted through a TGFβRI- and Smad2/3-dependent canonical and COX-2-dependent pathway. TGFβ3-induced signaling pathways are primarily associated with TGFβRI and TGFβRII. Once active TGFβ3 is released from a large latent TGFβ3 complex protein, it interacts with a heterotetrameric receptor complex carrying two TGFβRI subunits and two TβRII subunits [43,44]. It has been shown that Smad is a canonical pathway in which TGFβ is identified by TGFβRII equipped with an intracellular kinase domain [45]. Furthermore, the canonical TGFβ signaling pathway uses Smad2 and/or -3 to transfer signals, and Smad2/3 are directly phosphorylated by TGFβRI and then translocate to the nucleus to regulate gene transcription [46]. According to our findings, it was revealed that TGFβ3-induced COX-2 and TXB_2_ production could be blocked by TGFβRIi (LY364947, a TGFβRI inhibitor) and Smad3i (SIS3, a Smad3 inhibitor). In parallel, TGFβ3 directly caused Smad2/3 phosphorylation, and the phosphorylated Smad2/3 could be detected in the cell nucleus, as determined by cell fractionation, Western blot analysis, and immunofluorescence microscopy. Although PD98059 could also attenuate TGFβ3-induced TXB_2_ production, it did not affect COX-2 expression. Therefore, it is unlikely to involve the non-canonical TGFβ ERK/MAPK kinase signaling pathway in causing COX-2 expression but may instead include ERK1/2 in regulating the thromboxane secretion process in follicular GCs. 

PCR analysis to amplify the region of the COX-2 promoter has indicated possible Smad and other binding sites in the promoter region, including Smad binding elements (AGAC), one AP-1-like site (TGCGTGG), and one Sp1 site (GGGCGG) [47,48]. Moreover, TGFβ can regulate COX-2 expression in fibroblasts through multiple factors such as Smad2/3, in which the authors demonstrate in vivo binding of Smad2/3 to the COX-2 promoter and recruitment of Smad2/3 on the promoter by real-time PCR analysis [49]. These studies all demonstrate the existence of the Smad2/3 binding region within the COX-2 promoter region. That explains why, in this study, TGFβ3-promoted Smad2/3 phosphorylation and translocation to the nucleus could drive COX-2 transcription and protein induction in GCs. Interestingly, in human uterine stromal cells, TGFβ isoforms can also “downregulate” COX-2 expression in a Smad2/3-dependent manner, although the downregulation is indirect via upregulation of endoplasmic reticulum mannosidase I to enhance COX-2 degradation [50].

## 4. Materials and Methods

### 4.1. Materials

Human TGFβ3 was obtained from ProSpec-Tany TechnoGene Ltd. (East Brunswick, NJ, USA). PD98059, SB202190, SP600125, LY294002, SIS3, and actinomycin D were purchased from Sigma-Aldrich Chemical Co. (St. Louis, MO, USA). H-89 and GF109203X were obtained from Biomol (Farmingdale, NY, USA). The antibody (Ab) generated against phospho-Smad2/3 (#33102) was obtained from Cell Signaling Technology (Danvers, MA, USA). The Abs raised against total Smad2/3 (sc-11769) and lamin B (sc-6216) were obtained from Santa Cruz Biotechnology (Dallas, TX, USA). The Abs raised against total Smad2/3 (sc-11769) and lamin B (sc-6216) were obtained from Santa Cruz Biotechnology (Santa Cruz, CA, USA). The Ab raised against α-tubulin (GTX628802) was purchased from GeneTex (Irvine, CA, USA). TGFβ3 was dissolved in 4mM HCl-0.1% bovine serum albumin, which was used as a vehicle in this study.

### 4.2. Patient Recruitment and Ovarian FF Collection

The IVF patients were enrolled in this study. We analyzed an FF sample from a preovulatory leading (large) and mid-antral follicle (small) in each patient instead of pooling multiple FF samples from follicles. The human study was approved by the Ethics Committee of Cathay General Hospital, Taipei, Taiwan (CGH-P110098). Briefly, patients were treated by ovarian stimulation with a GnRH antagonist protocol and recruited as previously described [16]. During oocyte retrieval, two FF samples with one preovulatory leading follicle (follicle size > 18 mm) and one mid-antral follicle (follicle size < 14 mm) were collected from each IVF patient. Despite some oocytes being obtained during oocyte retrieval, only one FF sample was collected from the first retrieving procedure. The oocytes were evaluated based on their nuclear maturation status as previously described [51], which includes metaphase II (MII), metaphase I (MI), and germinal vesicle (GV) stages.

### 4.3. Preparation and Culture of Ovarian Follicular GCs

The human HO23 ovarian GC cell line was a kind gift from Dr. Tsai Eing-Mei (Kaohsiung Medical University, Kaohsiung, Taiwan). The 8–21 passages were used in this study. The primary cultured human ovarian follicular GCs were prepared from the FF of IVF patients receiving GnRH antagonist protocol. The isolation, preparation, and characterization of FF-containing GCs were performed by our laboratory [36]. In brief, FF was aspirated from patients undergoing oocyte retrieval for IVF. All of the follicular aspirates from each patient were mixed and centrifuged at 1000× *g* for 3 min, resuspended in phosphate-buffered saline (PBS) with 0.2% hyaluronidase, and incubated at 37 °C for 30 min. The suspension was layered onto Percoll (Sigma Aldrich, St. Louis, MO, USA) and centrifuged at 400× *g* for 30 min. The GCs were collected from the interphase, washed with PBS and seeded in tissue cultured flask, and maintained in M199 media supplemented with 10% FBS and 100 U/mL of penicillin, 100 μg/mL of streptomycin, and 25 μg/mL of amphotericin B (Thermo Fisher Scientific, Bohemia, NY, USA) at 37 °C. 

### 4.4. ELISA 

The level of TXB_2_ in the culture medium was measured by TXB_2_ ELISA kit (Cayman Chemical Company, Ann Arbor, MI, USA), whereas TGFβ3 in FF was determined by TGFβ3 ELISA kit (R&D Systems, McKinley Place, MN, USA). For determining TXB_2_ in the culture medium, the GCs (1 × 10^5^/well) were seeded in a 48-well culture plate for 24 h. After brief starvation overnight, cells were treated with the indicated concentrations of TGFβ3 for 24 h. At the end of incubation, the culture medium was collected and centrifuged, and the TXB_2_ levels were measured according to the manufacturer’s protocol. The enzymatic reaction produced a yellowish color that strongly absorbed light at 412 (TXB_2_) or 450 (TGFβ3) nm. For measuring TXB_2_ level, the intensity of this color was directly proportional to the amount of TXB_2_ tracer bound to the well and inversely proportional to the amount of free TXB_2_ present in the well during the incubation. The concentration of TXB_2_ in the GC culture medium and TGFβ3 in the FF were calculated from their respective standard curve.

### 4.5. Western Blot Analysis

GCs were harvested and lysed immediately using RIPA lysis buffer and then analyzed by SDS-PAGE. After being blotted onto PVDF membranes, the blots were hybridized with a specific primary antibody overnight at 4 °C. Subsequently, a secondary antibody-conjugated horseradish peroxidase was applied and incubated at room temperature for an additional 1 h. The immunoreactive bands were visualized using an enhanced chemiluminescence reagent (Immobilon Western Chemiluminescent HRP Substrate, EMD Millipore Corporation, Billerica, MA, USA). The luminescence images of the immunoblots were analyzed and acquired using Azure Biosystems (Azure Biosystems Inc., Dublin, CA, USA).

### 4.6. RT-PCR

The mRNA expression of COX-2, COX-1, and β-actin was determined by RT-PCR. Total RNAs of GCs were extracted using Trizol reagents (Invitrogen Life Technologies, Carlsbad, CA, USA), and a reverse transcription reaction was performed using the iScript cDNA Synthesis Kit (Bio-Rad Laboratories, Inc., Hercules, CA, USA). A total of 25 μL of the reaction mixture was prepared for the PCR reaction, containing cDNA, 2X master mix (GoTaq, Promega Corporation, Madison, WI, USA), primers, and nuclease-free water, and PCR was performed with a hot start at 94 °C for 5 min, followed by 30 cycles of denaturation at 94 °C for 1 min, annealing at 56 °C for 1 min, and elongation at 72 °C for 1.5 min. The amplification products were then analyzed by agarose gel electrophoresis in 1.5% agarose. The oligonucleotide primers for PCR targeting human COX-1, COX-2, and β-actin are listed in Table 2. 

### 4.7. Pharmacological Interventions

To dissect the role of the involved signaling molecule(s) in TGFβ3-induced TXB_2_ production, pharmacological interventions using some signaling inhibitors were performed. Briefly, the cells were treated with vehicle or TGFβ3 (10 ng/mL) in the presence of vehicle or the following inhibitors, including LY364947 for TGFβRI (designated as TGFβRIi), SIS3 for Smad3 (Smad3i), NS398 and indomethacin for COX-2 and/or COX-1 (COX-2i and COX1/2i), PD98059 for ERK1/2 (ERKi), SB2020190 for p38 MAPK (p38i), SP600125 for JNK1/2 (JNKi), and rapamycin for mTOR (mTORi) for the indicated time intervals. At the end of incubation, the culture media were collected, and cell lysates were prepared. The TXB_2_ content in cell media was analyzed by ELISA, whereas the effect of these signaling inhibitors on COX-2 expression was determined by Western blotting. The concentrations of each inhibitor were the following: COX-2i (NS-398): 10 nM; COX1/2i (indomethacin): 0.1 μM; TGFβRIi (LY364947): 1 μM; Smad3i (SIS3): 2 μM; ERKi (PD98059): 10 μM; p38i (SB2020190): 10 μM; PI-3Ki (LY294002): 10 μM; JNKi (SP600125): 10 μM; and mTORi (rapamycin): 1 μg/mL.

### 4.8. Cell Fractionation and Smad2/3 Translocation

The cytosolic and nuclear fractions of the cells were prepared using the NE-PER™ nuclear and cytoplasmic extraction reagents (Thermo Fisher Scientific, Waltham, MA, USA) according to the manufacturer’s protocol. The Smad2/3 translocation was determined by Western blot analysis of their distributions in the cytosolic and nuclear fractions.

### 4.9. Immunofluorescence Microscopy

Cells were washed, fixed with 4% paraformaldehyde (PAF) for 20 min, and permeabilized with 0.2% Triton X-100 for 10 min. Then, cells were blocked with 3% BSA, incubated with the Ab specific for Smad2/3 (1:250; Santa Cruz Biotechnology; goat anti-human type) at 4 °C overnight, and then followed by the anti-goat 2nd FITC-conjugated Ab (Chemicon) for an additional 1 h at RT. By the end of incubation, DAPI (1:1000; Thermo Fisher Scientific) was added to incubation for cell nucleus staining. After a brief wash, the tissue samples mounted on chamber slides were analyzed under a Nikon Eclipse Ti-S fluorescence microscope (Japan) and photographed using a digital camera.

### 4.10. SiRNA Interference

SiGenome control and COX-2 siRNAs were purchased from Dharmacon RNAi Technologies (Thermo Fisher Scientific), and a similar transfection assay was performed by our lab [52]. The cell cultures were transfected with control or COX-2 siRNAs (150 or 300 nM) for 24 h using the DharmaFECT transfection reagent. The cells and media were collected for further analysis.

### 4.11. Statistical Analysis

Otherwise indicated, data are presented as the mean ± standard error (SEM) and were analyzed by GraphPad Prism Software version is 10.2.3 (Boston, MA, USA). Data were compared using the two-tailed Student’s *t*-test. Statistical significance was considered as *p* < 0.05. The normality assumption of continuous variables was tested using the Shapiro–Wilk test. Data for non-normally distributed measures were assessed using the Mann–Whitney U test. For analyzing the correlation between FF TGFβ3 levels and oocyte maturity, the grading of oocyte maturity was performed by microscopic examination of oocytes’ morphology, and the FF TGFβ3 levels and oocyte maturity of all collected samples were examined by logistic regression and receiver operating characteristic (ROC) analysis. The area under the curve in ROC analysis was used to determine the probability of accurately distinguishing high-quality oocytes. These analyses were performed using SPSS version 18.0 (Chicago, IL, USA).

## 5. Conclusions

**Figure 8:**
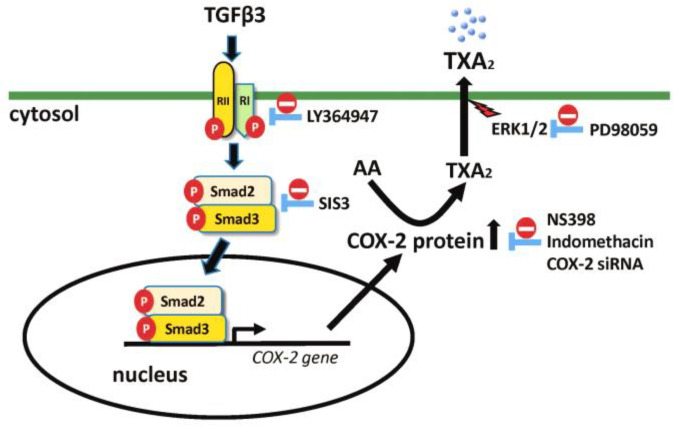
Proposed illustrated scheme for TGFβ3′s effects on follicular GCs. TGFβ3 binds TGFβ receptor II and I (RII and RI), leading to an activation of the canonical signaling pathway, including Smad2/3 phosphorylation and translocation to the cell nucleus. The activated Smad2/3 acts as a transcription factor to drive COX-2 mRNA and protein induction, which then triggers TXA_2_ generation and secretion into extracellular space. The 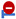 indicates a pharmacological and/or siRNA intervention used in this study.

We provided here the first evidence showing that TGFβ3 can provoke TX production in follicular GCs through a TGFβRI- and Smad2/3-dependent canonical and COX-2-dependent pathway. In addition, Erk1/2 may be involved in the TGFβ3-mediated TXB_2_ secretion process. The proposed illustrated scheme for TGFβ3′s effects on follicular GCs is presented in Figure 8. Our findings revealed the presence of TGFβ3 in human FF of IVF patients and its correlation with oocyte maturation. Our parallel study demonstrated the FF TX level can be used to predict oocyte maturity during IVF, and the TP receptor activation by a TXA_2_ analog can regulate follicle development in an ex vivo ovary culture [17]. This study delineated the function of TGFβ3 in ovarian FF and its ability and regulatory mechanism in causing FF TX production in GCs. These findings imply the possible roles of TGFβ3 in follicle development and oocyte maturation, which can be of value in the biology and endocrinology fields of female reproductive science.

## Figures and Tables

**Table 1:** Logistic regression analysis of TGFβ3 levels and oocyte maturity in mid-antral and preovulatory follicle groups.

Variable	Odds Ratio	95% C.I.	*p*-Value
TGFβ3	1.012	1.001/1.024	0.039 * (<0.05)

* *p* < 0.05.

**Table 2:** Primer sets for RT-PCR.

Gene	Forward Primer (5′-3′)	Reverse Primer (5′-3′)	Product Size (bp)
*COX-1*	ACATTCAGTTCCCACCATCT	TCACTGCTGTTGGGTCTCTG	601
*COX-2*	CAGCAAATCCTTGCTGTTCC	TGGGCAAAGAATGCAAACATC	517
*β-actin*	ATCATGTTTGAGACCTTCAA	CATCTCTTGCTCGAAGTCCA	314

## Data Availability

The data used to support the findings of this study will be provided upon request by the Journal.
